# Neural correlates of product attachment to cosmetics

**DOI:** 10.1038/s41598-021-03576-2

**Published:** 2021-12-20

**Authors:** Yoshiaki Kikuchi, Madoka Noriuchi, Hiroko Isobe, Maki Shirato, Naoyasu Hirao

**Affiliations:** 1 Department of Frontier Health Science, Graduate School of Human Health Science, Tokyo Metropolitan University, Tokyo, 116-8551 Japan; 2 Shiseido Global Innovation Center, Yokohama, 220-0011 Japan

**Keywords:** Neuroscience, Psychology

## Abstract

The neurobiological basis of brand and product attachment has received much attention in consumer neuroscience research, although it remains unclear. In this study, we conducted functional MRI experiments involving female users of famous luxury brand cosmetics as participants, based on the regions of interest involved in human attachment and object attachment. The results showed that the left ventral pallidum (VP), which is involved in positive reward, and the right posterior cingulate cortex (PCC), which is involved in self-concept, a key concept in object attachment, are the core regions in cosmetic attachment. Moreover, the performed psychophysiological interaction analyses showed that VP-temporoparietal junction connectivity positively correlated with activity in the dorsal raphe nucleus, and PCC–anterior hippocampus (aHC) connectivity positively correlated with subjective evaluation of attachment. The former suggests that object attachment is a human-like attachment and a stronger tendency of anthropomorphism is associated with stronger feelings of security. The latter suggests that the individual’s concept of attachment as well as the relationships with the attached cosmetics are represented in the aHC, and the PCC–aHC associations produce subjective awareness of the attachment relationships. These associations between memory and reward systems have been shown to play critical roles in cosmetic attachment.

## Introduction

Bowlby’s^1^ described attachment as the emotional bond between infants and their caregivers that is the foundation for further healthy development, and attachment theory as an inherent biobehavioral system to provide satisfaction of basic human needs. It has been suggested that attachments can be extended beyond the person-person relationship, and to the person-object relationship context in many studies on marketing^2–13^. Indeed, attachment has been suggested as the core of all strong brand relationships^14^, and the concept of attachment has been regarded as one of the most important concepts in the consumer brand relationship (CBR) in modern marketing literature. According to the definition by Park et al.^15^, attachment is a psychological state of mind in which a strong cognitive and affective bond connects a brand with an individual in such a way that the brand is viewed as an extension of the self. The personalized and affect-based representations of the connection are highly salient and automatically retrieved when consumers activate their self-concept^16^. According to the attachment theory^1^, security-providing interactions with attachment figures reinforce reliance on social support and construct positive working models of the self and others^17^. Similarly, consumers develop strong attachments to a brand when they believe it can be relied on^18–20^, and it becomes linked to the self when it is consistently trusted and felt a sense of security. In addition, it has been suggested that consumers view brands as possessing human characteristics^14,21,22^. Moreover, it was shown that a stronger anthropomorphism tendency was associated with enhanced perception of the objects’ sentimental and instrumental value, and this enhanced value mediated the relationship between anthropomorphism tendency and object attachment^23^. Therefore, it is important to examine how these characteristics of product/brand attachment are related to the brain activity and network in order to understand the neurobiological mechanisms of customers’ psychology and behaviors.

Based on the above facts and considerations, it should be validated whether these characteristics of brand/product attachment^18–23^, as well as its definition by Park et al.^15^, could be explained based on the neurobiological basis. However, this remains unclear. Recently, a study^24^ has shown that oxytocin (OXT), which is a central neuropeptide in the formation and maintenance of human and animal attachment, increases following exposure to one’s favorite brand. In addition, this study showed positive associations between baseline peripheral OXT concentrations and brand relationship quality^24^. However, it is difficult to clarify the neural basis of attachment to general products or brands, because there are so many kinds of products or brands. Despite such a constraint, this study clearly provided a neurochemical basis for object attachment, which is common to human attachment, in the categories of food, beverages, and body care products^24^. Therefore, this study established a milestone toward developing neuroscientific approaches to understand the neural network underlying object attachment, including brands/products. Thus, we examined the neural basis of object attachment by using face care products of famous brands in the same category of skin care products in cosmetics as in the above study^24^. Cosmetic users use their favorite cosmetics to maintain their own beauty and health (“secure state for the self”) or to become closer to realizing an ideal vision of themselves (“ideal self”). This fact suggests that self-concept is also a key concept in cosmetic attachment, as in general brands/products^15^.

Based on these facts and considerations, we first hypothesized the involvement of the reward system, which has been shown to be involved in human attachment and animal pair bonding. The ventral pallidum (VP) has been shown to be particularly important in the brain regions of the reward system. Animal experiments have shown that activity in the VP is linked with pair bonding and attachment behaviors in monogamous prairie voles^25,26^. In humans, securely attached children show greater VP activation than children with poor attachment^27^. In addition, the VP shows significant activities in both maternal love^28^ and romantic love^29^. Furthermore, similar to human attachment, a recent neuroimaging study related to decision making in hoarding disorders showed that the VP is activated in association with attachment to object possessions^30^, suggesting that the VP plays an important role not only in attachment in individuals, but also in object attachment. Second, we hypothesized the involvement of neural processes for self-referential information and autobiographical and conceptual (social) memories, because self-concept is a key concept in object attachments, including brands and products. In addition, it is suggested that self-extension processes personalize (index) particular material objects with autobiographical meanings^31^, endowing them with personal meanings that connect the self and object^2^. Therefore, the brain regions involved in self-referential processing and autobiographical memory, including the posterior cingulate cortex (PCC), retrosplenial cortex (RSC), hippocampus (HC), and temporoparietal junction (TPJ), would play important roles in object attachment^32^. Specifically, the anterior HC, which represents the conceptual (semantic) memory, is involved in indexical (personalized) representations of the attached object as part of the self-concept. Third, we hypothesized that the brain regions including the TPJ, which is involved in social cognition^33,34^ and anthropomorphism^35^, would play an important role in object attachment, because stronger anthropomorphism is associated with stronger object attachment. In addition, the neural basis of object attachment is based on the mutual communications among the brain regions related to the reward, self-referential processing, autobiographical memories, social cognition, and anthropomorphic processes. Such a neural basis would be involved in self-awareness and a sense of security related to object attachment. Our participants were regular (loyal) users of the face serum of a famous luxury brand. The functional magnetic resonance imaging (fMRI) measurements were performed when the participants (1) viewed individual photos of their attached and non-attached cosmetic bottles, and (2) viewed each cosmetic bottle photo while their left hand was gently massaged by a beauty specialist using the serum. The latter setting was based on the consideration that the daily self-touching behaviors may reinforce the bond between the users and their favorite cosmetics, and this intimate relationship may establish a specific and stable attachment to the cosmetic product, as in human attachment in relationships. In addition, there is the possibility that touching behaviors such as massage facilitate OXT release from the hypothalamus^36–39^, which facilitates neural activation in the neural network underlying object attachment, and these neural processes lead to heightening the detectability of fMRI signals. Furthermore, we set the regions of interest (ROIs) based on the brain regions identified in previous fMRI studies of human attachment in relationships^29,40–43^ (Table 1) and of attachment to object possession^30^. Each differential ROI activity between the attached and non-attached cosmetics was tested using the small volume correction (SVC) test in the visual and visual with tactile sessions. In addition, we performed an analysis of variance (ANOVA) for the ROI activities that were retained after the SVC test and the VP ROI activity that was hypothesized to be a key in attachment relative to baseline. Thereafter, we performed psychophysiological interaction (PPI) analyses using the ROI activities, which showed a significant main effect of attachment and the interactive effect of touch and attachment, as the seeds for analyses. In addition, we investigated the correlation of individual strength of connectivity with subjective evaluation and brainstem ROI activities.

**Table 1:** Summary of the previous fMRI studies of human attachment relationships.

fMRI study	Numbers and age of subjects	Brain regions involved in reward and memory, and the brainstem regions	Duration of relationships
Aron et al.^29^	17 subjects (10 females, 7 males) 18–26 years (mean = 20.6 years, median = 21 years)	Substantia nigra/ventral tegmental area, Nucleus accumbens/ventral striatum, Posterior hippocampus	1–17 months, mean = 7 months, median = 7 months
Bartels and Zeki^41^	17 subjects (11 females, 6 males) 21–37 years (mean = 24.5 years, median = 23 years)	Posterior hippocampus	mean = 2.4 years, s.d. = 1.7 years, median = 2.3 years
Kikuchi et al.^42^	17 subjects (males) 22–43 years (meadn = 31.4 years, s.d. = 7.7 years)	Posterior cingulate cortex, Dorsal raphe nucleus, Lateral coeruleus, Periaqueductal grey	11 subjects married (marital duration = 5.2 ± 5.9 years, age; 35.0 ± 6.8 years) 6 subjects not married (relation duration = 1.7 ± 1.3 years, age 24.8 ± 3.7 years)
Acevedo et al.^40^	17 subjects (10 females, 7 males) 39–67 years (mean = 52.9 years, s.d = 8.9 years)	Substantia nigra/ventral tegmental area, Globus pallidus, Posterior cingulate cortex, Posterior hipocampus, Dorsal raphe nucleus	All married 10–29 years, mean = 21 years, s.d = 5.9 years

## Results

**Figure 1:**
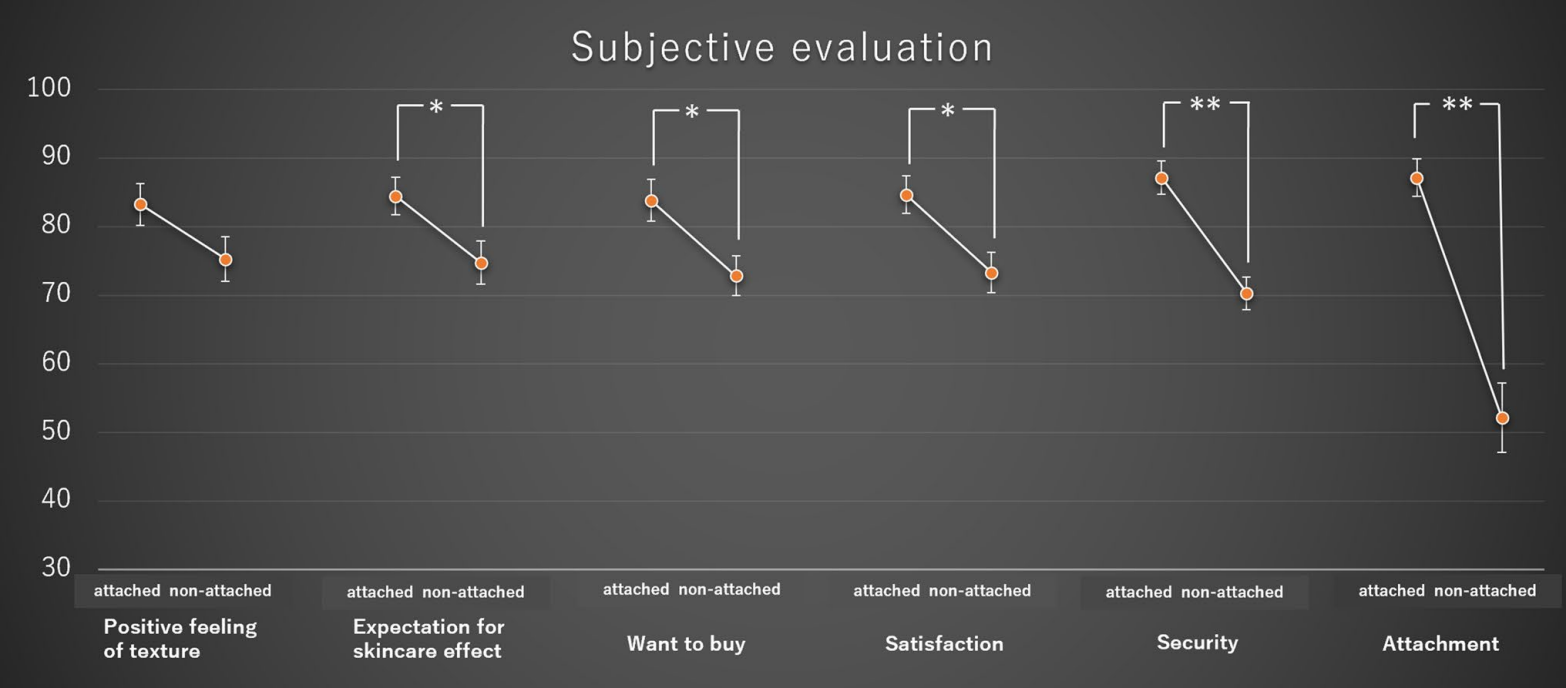
Comparisons of subjective evaluations between attached cosmetics versus non-attached cosmetics for each of the six items. *represents significance as assessed by a paired t-test (*p* < 0.05), and **represents significance as assessed by a paired t-test (*p* < 0.01).

Among the attached and non-attached cosmetics, the subjective evaluation scores showed significant differences for “security” (df = 9, t = 5.2, *p* = 0.053 × 10^−3^), “attachment” (df = 9,t = 5.9, *p* = 0.010 × 10^−3^), “expectation for skincare effect” (df = 9,t = 2.2, *p* = 0.044), “want to buy” (df = 9, t = 2.5, *p* = 0.022), and “satisfaction” (df = 9, t = 2.7, *p* = 0.014), whereas “positive feeling of texture” was not significant (df = 9, t = 1.8, *p* = 0.097) (Fig. 1).

**Figure 2:**
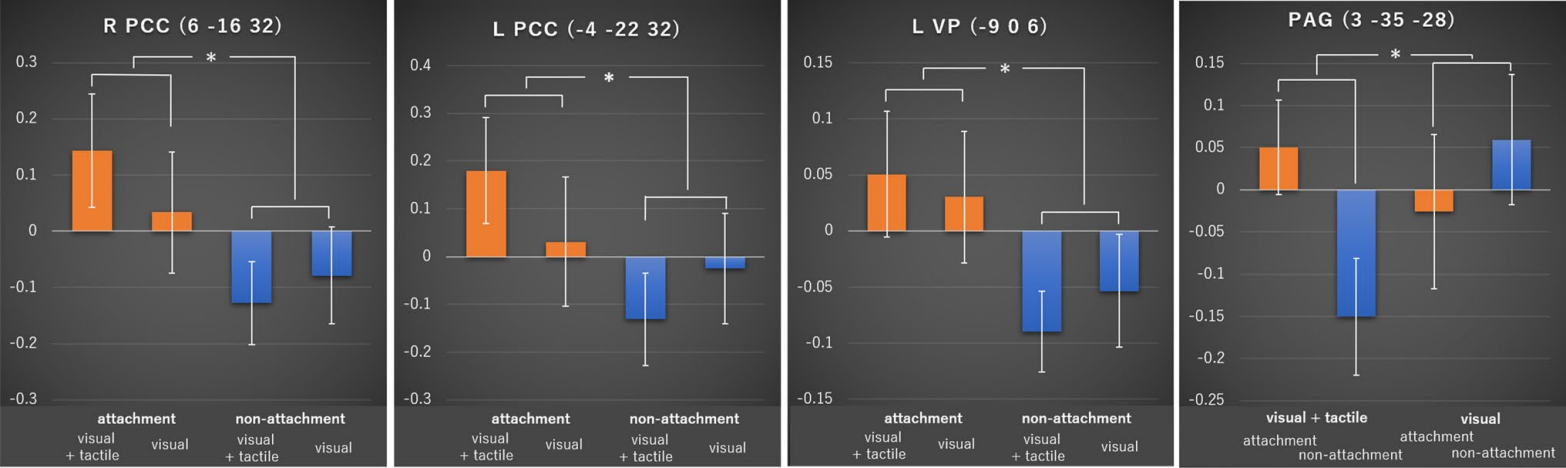
Results of the ANOVA. The left posterior cingulate cortex (PCC; − 4, − 22, 32), right PCC (6, − 16, 32), and left ventral pallidum (VP; − 9, 0, 6) demonstrated the main effect of attachment. The PAG (3, − 35, − 28) showed an interactive effect. *p* < 0.05. PCC, posterior cingulate cortex; VP, ventral pallidum; PAG, periaqueductal grey.

In the visual with tactile session, the ROI analyses showed that the right PCC (Montreal Neurological Institute (MNI) coordinates: 3, − 22, 29; family-wise error rate [pFWE] = 0.018^18,20^), right posterior HC (39, − 31, − 8; pFWE = 0.031^18^), left posterior HC (− 40, − 36, − 12; pFWE = 0.027^20^), right posterior HC (40, − 31, − 4; pFWE = 0.021^20^), right putamen (29, 2, − 3; pFWE = 0.028^20^), left PCC (− 4, − 22, 32; pFWE = 0.025^34^), right PCC (6, − 16, 32; pFWE = 0.090 × 10^−1^ ^34^), dorsal raphe nucleus (DRN; 6, − 32, − 24; pFWE = 0.041^19,34^), substantia nigra/ventral tegmental area (SN/VTA; 4, − 20, − 16; pFWE = 0.047^19^), periaqueductal gray (PAG; 3, − 35, − 28; pFWE = 0.034^21,34^), right middle insula (42, − 4, 2; pFWE = 0.049^19^), temporal gyrus (46, 2, − 10; pFWE = 0.013^19^), left angular gyrus (− 64, − 48, 26; pFWE = 0.030^19^), right middle frontal gyrus (30, 18, 57; pFWE = 0.038^30^), left inferior frontal gyrus (− 39, 33, 0; pFWE = 0.043^30^), insular cortex (− 36, 15, − 3; pFWE = 0.020^30^), middle cingulate gyrus (− 3, − 33, 39; pFWE = 0.045^30^), and anterior cerebellum (0, − 63, − 30; pFWE = 0.031^30^) were significantly activated in the attached cosmetic compared to that in the non-attached cosmetics, as shown in Table 2. In contrast, there were no significant differences in activity during the visual session. The ANOVA showed that there were significant main effects of attachment in the right PCC (6, − 16, 32; F = 10.443, *p* = 0.004), left PCC (− 4, − 22, 32; F = 5.791, *p* = 0.026), and left VP (− 9, 0, 6; F = 9.10; *p* = 0.007), all of which showed significantly greater activity in the attached than in non-attached cosmetics (Fig. 2). In addition, the PAG (3, − 35, − 28) was found to have a significant interactive effect between attachment and touch (F = 5.6; *p* = 0.029), and also had significantly greater activity in the attached than in the non-attached cosmetics, in the visual with tactile session (df = 9, t = 2.7; *p* = 0.014 < 0.025 = 0.05/2) (Fig. 2), while there was no significant difference in the visual session.

**Table 2:** Differential activity between the attached versus non-attached cosmetics.

L/R	Brain region	MNI coordinates			Visual		Visual + tactile		fMRI study (ROIs)
		x	y	z	pFWE	T	pFWE	T	
R	PCC	3	− 22	29	0.375	0.92	0.018*	3.24	^29^(7 months); ^41^(2.4 years)
	Posterior hippocampus	39	− 31	− 8	0.464	0.56	0.031*	2.92	Aron et al.^29^ (7 months)
R	Putamen	29	2	− 3	0.175	1.73	0.028*	2.99	Bartels and Zeki^41^ (2.4 years)
L	Posterior hippocampus	− 40	− 36	− 12	0.322	1.12	0.027*	3.02	
R	Posterior hippocampus	40	− 31	− 4	0.464	0.56	0.021*	3.16	
R	PAG	3	− 35	− 28	0.599	− 0.25	0.034*	2.86	Bartels et al. (2004) (maternal love)^42^; (5.6 years)
L	PCC	− 4	− 22	32	0.439	0.66	0.025*	3.06	Kikuchi et al.^42^ (5.6 years)
R	PCC	6	− 16	32	0.311	1.16	0.009*	3.61	
R	DRN	6	− 32	− 24	0.575	− 0.08	0.041*	2.76	
R	SN/VTA	4	− 20	− 16	0.364	0.96	0.047*	2.67	Acevedo et al. (2011) (21.5 years)
	Middle insula	42	− 4	2	0.241	1.44	0.049*	2.65	
	Temporal gyrus	46	2	− 10	0.501	0.38	0.013*	3.43	
L	Angular gyrus	− 64	− 48	26	0.433	0.69	0.030*	2.95	
R	Middle frontal gyrus	30	18	57	0.533	0.20	0.038*	2.80	Tolin et al.^30^ (object possession)
L	Inferior frontal gyrus	− 39	33	0	0.440	0.66	0.043*	2.73	
	Anterior insula	− 36	15	− 3	0.411	0.78	0.020*	3.17	
	Middle cingulate cortex	− 3	− 33	39	0.407	0.79	0.045*	2.71	
	Anterior cerebellum	0	− 63	− 30	0.481	0.48	0.031*	2.93	

Significant probability (pFWE) and T value of each ROI activity are shown in each session (SVC, *p* < 0.05 FWE).

**Figure 3:**
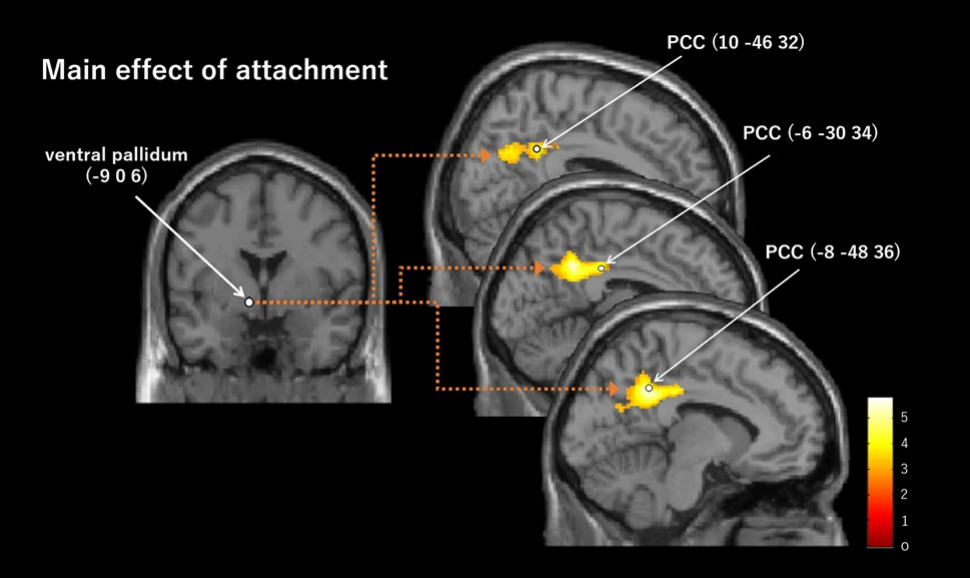
The left VP (− 9, 0, 6) showed a significant positive connectivity with several regions in the PCC ([− 8, − 48, 36], [− 6, − 30, 34], and [10, − 46, 32]) in the main effect of attachment. PCC, posterior cingulate cortex; VP, ventral pallidum.

**Figure 4:**
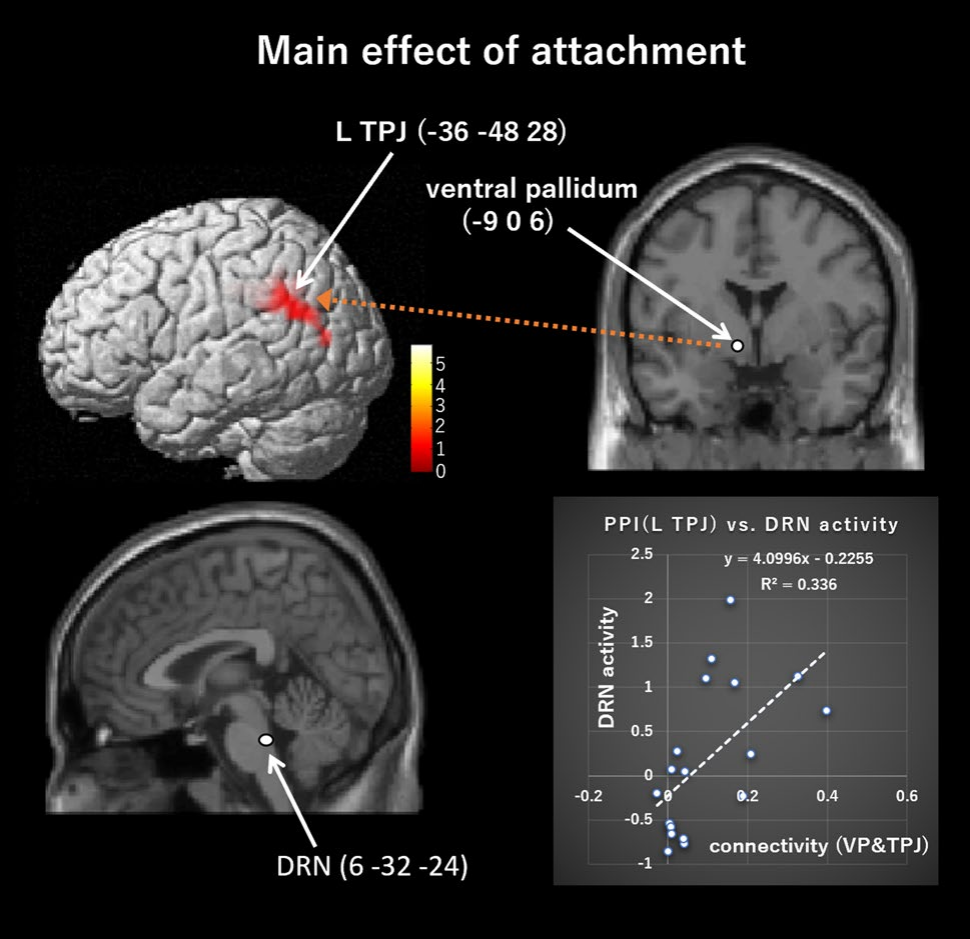
A positive connectivity between the left VP (− 9, 0, 6) and the left temporoparietal junction (TPJ; − 36, − 48, 28) was observed in the main effect of attachment, and its individual strength positively correlated with individual activity in the dorsal raphe nucleus (DRN; 6, − 32, − 24).

**Figure 5:**
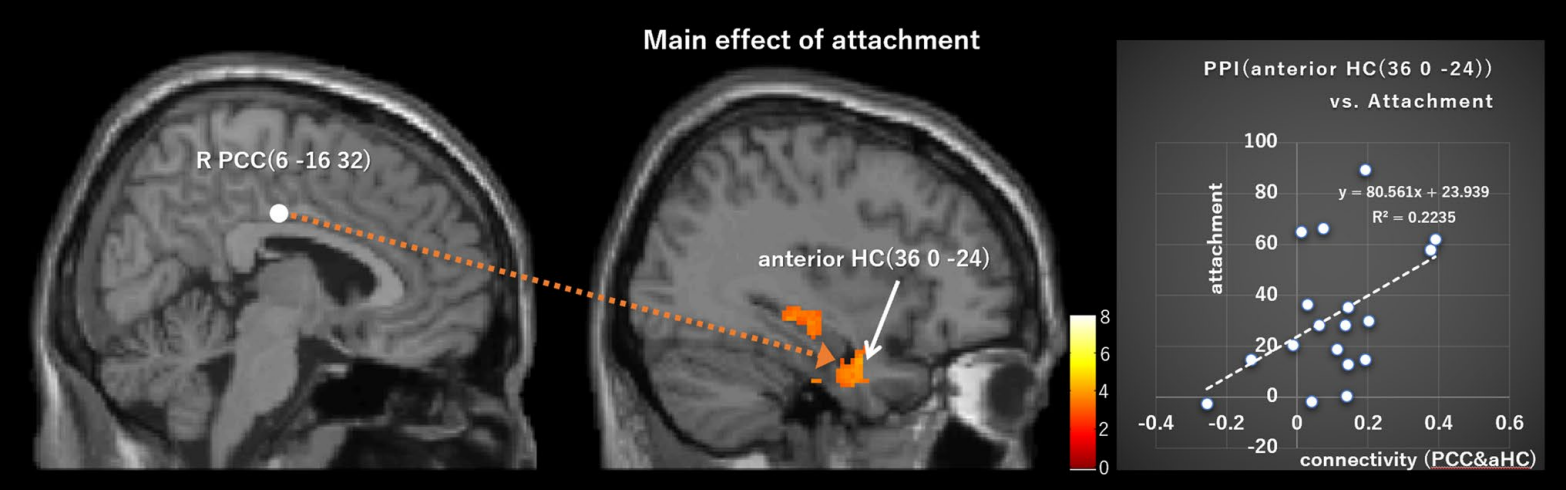
The right PCC (6, − 16, 32) showed a positive connectivity with the right anterior hippocampus (HC; 36, 0, − 24) in the main effect of attachment. The individual strength of connectivity between the right PCC (6, − 16, 32) and anterior HC (36, 0, − 24) in the main effect of attachment positively correlated with individual subjective awareness of “attachment.”

**Figure 6:**
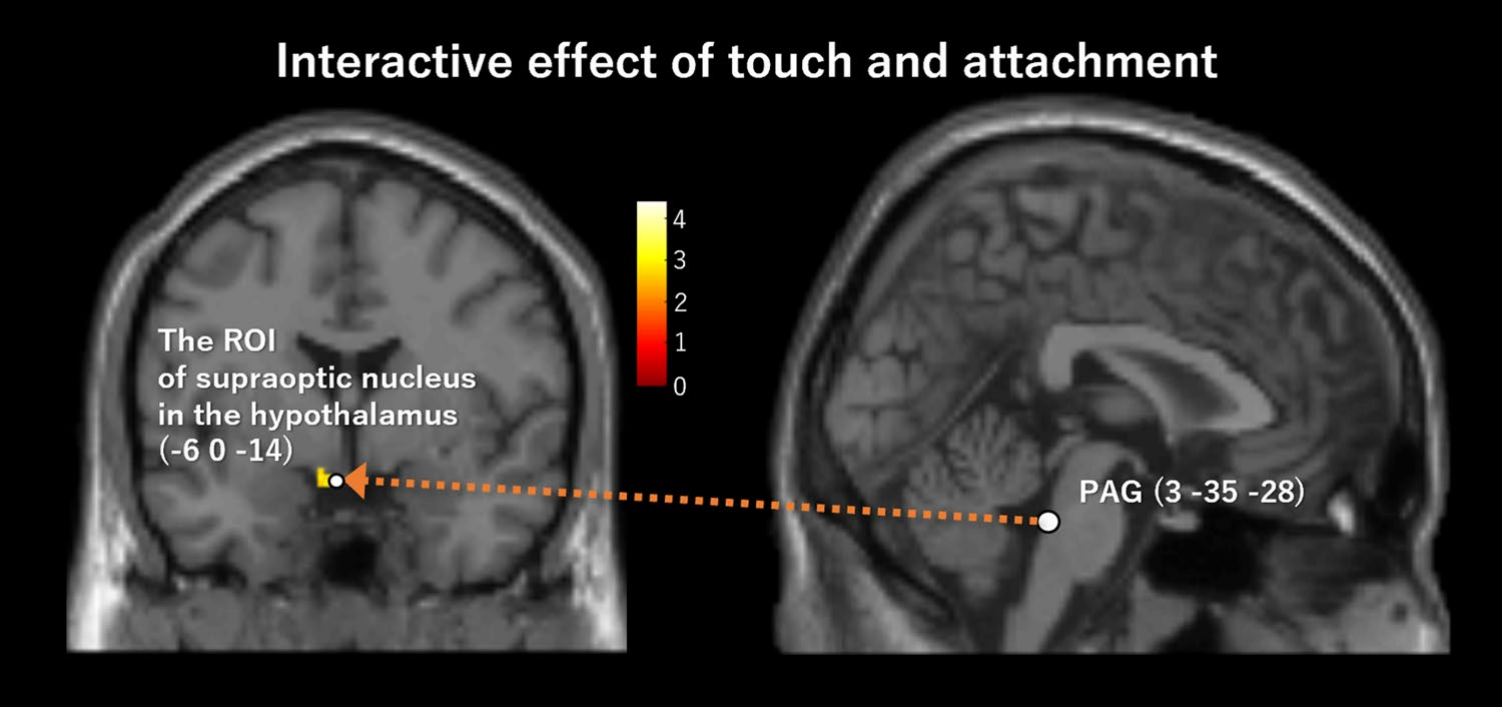
The right PAG (3, − 35, − 28) showed a positive connectivity with the ROI of right supraoptic nucleus in the hypothalamus (− 6, 0, − 14) in the interactive effect of touch and attachment.

The PPI analyses (peak *p* = 0.005, cluster-level pFWE < 0.05, height threshold T = 2.90, extent threshold = 501 voxels, df = [1.0, 19.0]; peak *p* = 0.001, cluster-level pFWE < 0.05, height threshold T = 3.65, extent threshold = 367 voxels, df = [1.0, 19.0]) showed that the left VP (− 9, 0, 6) positively connected to the left PCC ([− 8, − 48, 36], [− 6, − 30, 34]; peak *p* = 0.005, cluster-level pFWE = 0.000, cluster size = 1447 voxels; peak *p* = 0.001, cluster-level pFWE = 0.001, cluster size = 367 voxels), right PCC (10, − 46, 32; peak *p* = 0.005, cluster-level pFWE = 0.000, cluster size = 1447 voxels) (Table 3, Fig. 3), and left temporoparietal junction (TPJ; [− 36, − 48, 28], [− 52, − 50, 34], [− 48, − 74, 12]; peak *p* = 0.005, cluster-level pFWE = 0.030, cluster size = 501 voxels) (Table 3, Fig. 4), in the main effect of attachment. Moreover, the PPI analyses (peak *p* = 0.005, cluster-level pFWE < 0.05, height threshold T = 2.09, extent threshold = 484 voxels, df = [1.0, 19.0]; peak *p* = 0.001, cluster-level pFWE < 0.05, height threshold T = 3.65, extent threshold = 175 voxels, df = [1.0, 19.0]) showed that the right PCC (6, − 16, 32) positively connected to the left anterior HC ([− 32, − 6, 28], [− 44, − 2, − 34], [− 24, − 10, − 30]; peak *p* = 0.005, cluster-level pFWE = 0.009, cluster size = 634 voxels; peak *p* = 0.001, cluster-level pFWE = 0.005, cluster size = 290 voxels), right posterior HC ([40, − 22, − 10], [42, − 22, − 10] ), right anterior HC (36, 0, − 24; peak *p* = 0.005, cluster-level pFWE = 0.035, cluster size = 484 voxels) (Fig. 5), left cerebellar hemisphere (lobule V/VI; [− 38, − 50, − 34], [− 42, − 60, − 28], [− 46, − 52, − 32]; peak *p* = 0.005, cluster-level pFWE = 0.009, cluster size = 634), left pulvinar (− 22, − 32, 4), left thalamus (− 2, − 16, 10), right retrosplenial cortex (RSC; 2, − 50, 2) (peak *p* = 0.005, cluster-level pFWE = 0.001, cluster size = 870 voxels), and right thalamus (− 16, − 24, 10) (peak *p* = 0.001, cluster-level pFWE = 0.047, cluster size = 175 voxels), in the main effect of attachment (Table 3). There was no significant effective connectivity with the left PCC (− 4, − 22, 32). In addition, there was no significant connectivity with PAG (3, − 35, − 38) in the interactive effect of attachment and touch. As for the connectivity with PAG, we additionally set the supraoptic area (− 6.1, 0.5, − 16.0) in the hypothalamus^44^ as the ROI (r = 2 mm), because the PAG receives oxytocinergic fibers from the supraoptic nucleus in the hypothalamus^45^. This analysis showed a significant connectivity to this ROI (− 6, 0, − 14) in the interactive effect of attachment and touch (pFWE = 0.016 < 0.05, SVC) (Fig. 6).

**Table 3:** Results of the PPI analysis with each of the left ventral pallidum and the right posterior cingulate cortex as a seed.

L/R	Brain region	MNI coordinates			( ): peak *p* = 0.001 and cluster-level pFWE < 0.05		
		x	y	z	Cluster size	Cluster-level pFWE	T (peak-level)
**PPI (left ventral pallidum)**, Main effect; Cluster-level pFWE < 0.05 (peak *p* = 0.005, cluster > 501; *peak *p* = 0.001, cluster > 367).							
L	PCC*	− 8	− 48	36	1447 (367)	0 (0.001)	5.81
	PCC*	− 6	− 30	34			4.66
R	PCC	10	− 46	32			4.45
L	TPJ	− 36	− 48	28	501	0.03	3.95
		− 52	− 50	34			3.87
		− 48	− 74	12			3.77
**PPI (right posterior cingulate cortex)**, Main effect; Cluster-level pFWE < 0.05 (peak *p* = 0.005, cluster > 484; *peak *p* = 0.001, cluster > 175).							
L	Anterior HC*	− 32	− 6	− 28	634 (290)	0.009 (0.005)	7.97
		− 44	− 2	− 34			4.69
		− 24	− 10	− 30			4.42
R	Posterior HC	40	− 22	− 10	484	0.035	5.59
		42	− 22	− 10			4.3
R	Anterior HC	36	0	− 24			4.09
L	Cerebellum hemisphere	− 38	− 50	− 34	634	0.009	5.15
		− 42	− 60	− 28			5
		− 46	− 52	− 32			4.92
L	Pulvinar*	− 22	− 32	4	870 (175)	0.001 (0.047)	5.02
L	Thalamus	− 2	− 16	10			4.75
R	Retrosplenial cortex	2	− 50	2			4.63
R	Thalamus*	− 16	− 24	10	175	0.047	4.59

Multiple regression analyses showed that the individual strength of connectivity of the right PCC (6, − 16, 32) to the right anterior HC (36, 0, − 24) in the main effect of attachment positively correlated with “attachment” (df = 13, t = 2.150, *p* = 0.048; R^2^ = 0.175; Durbin-Watson [D-W] statistic = 1.921; Kolmogorov–Smirnov test *p* = 0.200) (Fig. 5). In addition, the individual strength of connectivity between the left VP (− 9. 0, 6) and left TPJ (− 36, − 48, 28) in the main effect of attachment positively correlated with the DRN (6, − 32, − 24) activity (df = 16, t = 2.845, *p* = 0.012; R^2^ = 0.294; D-W statistic = 1.580; Kolmogorov–Smirnov test *p* = 0.200) (Fig. 4).

## Discussion

Analysis of the subjective evaluations showed that the score of “attachment” was significantly higher for the attached cosmetics than for the non-attached ones, and it was confirmed that our participants felt a stronger attachment to their attached cosmetics than to the others. In addition, positive emotion or motivation including “security” was significantly higher for the attached cosmetics than for the non-attached ones. As for the brain regions involved in attachment to the cosmetics, the left VP and the left and right PCC were significantly activated in the main effect of attachment. Specifically, the left VP and right PCC showed significant functional connectivity to several other brain regions that are important for object attachment. These results show that these two regions play a major role in cosmetic attachment. Each of the VP and PCC is well known to be a core region in the reward system^46^ and self-referential processing system^47^, respectively. The VP is a central convergent region for input from the orbitofrontal, prefrontal, and infralimbic cortex, amygdala, lateral hypothalamus, VTA, parabrachial nucleus, subthalamic nucleus, and other structures related to reward^48^. Conversely, the VP projects back to nearly all of its input sources, including the nucleus accumbens for reciprocal information exchange^49^. Based on such limbic-related anatomical connectivity, the VP mediates reward and motivation functions at many levels in the brain^50,51^, such as social affiliation and pair bonding^52–55^. Indeed, activity in the VP is well known to be linked with pair bonding and attachment behaviors in monogamous prairie voles^25^. Moreover, securely attached children show greater VP activation than children with poor attachment^27^. In addition, the VP shows significant activities in both maternal love^28^ and romantic love^29^. Fisher et al.^56^ speculated that human activity in this region is likely related to feelings of attachment. Furthermore, a recent neuroimaging study related to decision making in hoarding disorders showed that the VP is activated in association with attachment to object possessions^30^. These findings and our present results show that the VP plays an important role not only in attachment in individuals, but also in object attachment, as we hypothesized. In addition, the PCC is the core region not only in the self-referential processing system^32^, but also in the processing systems of autobiographical memory^57^ and personal semantic memory (PSM)^47,58^. PSM is an intermediate entity between semantic and episodic memory^59^. Moreover, the PCC is well known as the strongest hub, with the highest number of functional connections^60^, and the core region of the default mode network (DMN) which is involved in self-referential processing^57^. Furthermore, this region plays a central role in supporting internally directed attention and cognition^60^. Based on these facts, the PCC region may be highly involved in self-concept, which is a key concept of object attachment^15,16^. Moreover, the PCC plays a critical role for craving^61^, and its activity represents the situations that the individual is “caught up” or “attached to” his/her experiences^62^. In addition, it has been recently shown that a substantial part of the serotonergic influence on this core region in the DMN is mediated by different 5-HT1A binding sites^63^. This fact suggests the relationship between the PCC activity and the subjective feelings of “security” which is one of the most critical emotions in brand/product attachment^18–20^.

Moreover, the left VP showed significant connectivity to several regions in the right PCC, and the left TPJ, and the PCC showed significant connectivity to the anterior HC and the RSC in the main effects of attachment. All these brain regions are included in neural networks for processing autobiographical and personal semantic memories^64^. Personal semantics are autobiographical knowledge or information extracted from repeated autobiographical events and is thought to be an intermediate entity between semantic memory and episodic memory^64^. Accordingly, such memory processing-related networks are considered to play a critical role in cosmetic attachment. Furthermore, the VP–PCC connectivity suggests that the association between neural networks for processing reward-related information and memory- and self-related information is the core mechanism for cosmetic attachment. Interestingly, such memory and reward associations have also been shown to be core mechanisms in nostalgic experiences^65,66^. Moreover, it has been shown that greater nostalgic connection between customers and products link to greater intensity of product attachment in marketing literature^13,67–70^. In addition, there was a significant positive connectivity between the left VP and left TPJ in the main effect of attachment, and its individual strength was positively correlated with individual DRN activity (Fig. 4). The TPJ is well known to be involved in social cognition and mentalizing^71^. In particular, the left TPJ is involved in processing different subjective perspectives^72^. This suggests that the participant might view her attached cosmetic as possessing human characteristics. Indeed, it has been shown that there is an association between the structure of the left TPJ and anthropomorphism^35^. Fournier^14^ claimed that individuals experience little difficulty in assigning personality features to brands, and it has been shown that consumers easily view brands as possessing human characteristics^21,22^. Moreover, Kwok et al.^23^ showed that a stronger anthropomorphism tendency was associated with enhanced perception of the objects’ sentimental and instrumental value, and this enhanced value mediated the relationship between anthropomorphism tendency and object attachment. In the present study, it was shown that a stronger anthropomorphism tendency represented in the left TPJ was associated with a higher reward value of the attached cosmetic represented in the left VP. Therefore, anthropomorphism was shown to play a critical role in cosmetic attachment, as hypothesized. Furthermore, our present results showed that stronger connectivity between the left VP and TPJ regions induces greater activity in the DRN. This result suggests that individuals who showed stronger connectivity between these regions feel more secure feelings because the DRN is a serotonin-rich site. Furthermore, our previous studies^42,43^ showed that DRN activity is negatively correlated with attachment-related anxiety. Therefore, it is suggested that individuals with less attachment-related anxiety, that is, those who have better attachment relationships with the objects, show stronger neural communication between the VP and the TPJ, and assign personality features to the object (anthropomorphism). This result showed that the left VP and the left TPJ might coproduce a sense of security, which is one of the critical factors in product/brand attachment^18–20^, as hypothesized. In addition, our results of the left TPJ-VP connectivity may be based on similar mechanisms in a recent neuroimaging study that showed a similar relationship between functional connectivity between the left TPJ and ventral striatum and the positive feelings such as happiness^67,68^.

Moreover, there was positive connectivity between the right PCC and the right anterior HC, and the individual strength of this connectivity positively correlated with the individual subjective score of “attachment” (Fig. 5). The anterior HC region is involved in categorical/conceptual representations, while the posterior region is associated with the recovery of fine-grain perceptual detail^73^. Accordingly, the anterior HC is considered to be involved in establishing one’s concept of attachment relationships with cosmetics by combining multiple forms of information, such as semantic and emotional information^74^, or integrating distinct experiences on a conceptual scale^75^. In addition, genetic and pharmacological studies have revealed that OXT receptors in the anterior dentate gyrus and CA2/CA3 play a critical role in the discrimination of social stimuli, and OXT receptors in the anterior CA2/CA3 neurons recruit a population-based coding mechanism to mediate social stimuli discrimination^76^. It has been suggested that there are indexical (personalized) and affect-based representations of the brand as part of the consumer’s self-concept in strong connections between customers and products. Thus, such representations are highly salient and automatically retrieved when the consumer activates his or her self-concept^16^. Such mental representations may be schemata that encode past experiences of the interactions with the attached object, and of its availability and capacity to respond to the user’s needs. The present results suggest that such schemata are represented in the anterior HC and make it possible to discriminate one’s attached cosmetic as the special entity for her from the other cosmetics. Furthermore, from the perspective of social memory, it might discriminate one’s attached cosmetic, which is like the person close to her (anthropomorphism) from the others. Accordingly, the PCC–anterior HC connectivity suggests that the sensory stimulation cues induce the automatic retrieval of such mental representations in the anterior HC, and the individual re-experiences the autobiographical episodes associated with such representations, mediated via the PCC–anterior HC connectivity, and these neural processes lead to one’s subjective awareness of attachment to the cosmetic.

In addition, there was a significant interaction in the PAG activities, and the PAG showed greater activation for attached cosmetics in the visual with tactile session, while it showed a greater deactivation for non-attached cosmetics. This structure is heavily connected to various limbic regions and contains a high density of OXT receptors^77^. OXT is implicated in regulating positive social interactions, social bonding, and maternal responsiveness in several mammalian species, including humans^78^. It has been shown that intranasal delivery of synthetic OXT motivated pair-bonded men to maintain a larger social distance from an unknown female experimenter and inhibited approach toward attractive women^79^, suggesting that OXT is also important for the maintenance of an already established pair bond^80^. Accordingly, this interactive effect found in PAG activities may be explained by a similar mechanism of approaching or avoiding behavior, based on the effects of OXT on the PAG. In addition, the PAG receives oxytocinergic fibers from the supraoptic nucleus in the hypothalamus^81–84^. Our additional analysis showed significant functional connectivity between the PAG and supraoptic nucleus in the hypothalamus (Fig. 6). Moreover, upregulation of OXT expression in the hypothalamus is known to be activated by somatosensory stimulation, such as massage, which is mediated via the spinothalamic pathway^39^. The thinly myelinated or unmyelinated afferent fibers activated by touching or rubbing are carried by the contralateral spinothalamic pathway. These impulses are sent to the thalamus and then the primary somatosensory cortex. These impulses are further sent to other brain regions, including the PAG, hypothalamus, and brainstem, via collateral connections^85^. Accordingly, the interactive effect observed in the PAG activity in the present study may also be explained by such a synergetic effect between direct somatosensory stimulation and OXT release via hypothalamic activity. In addition, several ROI regions were significantly activated in the visual with tactile session. However, there was no significant activity in these regions during the visual session. These brain regions include the memory-, reward-, emotion-related regions, and brainstem regions such as the DRN, PAG, and SN/VTA, which are the primary sites of neurotransmitters that modulate the basic functions of survival, such as reward, motivation, emotion, and security^42,43^. These results may also highlight the importance of tactile information processing in attachment to cosmetics. Skin-to-skin contact is one of the earliest communication channels that promotes attachment between infants and caregivers^84^. The OXT release associated with tactile stimulation, via hypothalamic activity, was considered to have some effects on these several brain regions involved in object attachment.

In the present study, the results showed that the left VP involved in positive rewards and the right PCC involved in the self-concept which is a key concept in object attachment, are the core regions in cosmetic attachment. Moreover, the associations between the reward (VP) and memory (PCC) systems were shown to play critical roles in cosmetic attachment. Furthermore, the PPI analyses showed that the VP-TPJ connectivity positively correlated with activity in the DRN, and the PCC–anterior HC connectivity positively correlated with subjective evaluation of attachment. The former suggests that object attachment is a human-like attachment and a stronger tendency of anthropomorphism is associated with stronger feelings of security. The latter suggests that the individual’s concept of attachment as well as the relationships with the attached cosmetics is represented in the aHC, and the PCC–aHC associations produce the subjective awareness of the attachment relationships. However, some limitations of the present study should be noted. First, the research focuses on only one product category (i.e., face care products). Here, we showed the important concepts and characteristics of object attachment are based on the neural network centered on the VP and PCC and their functional connectivity. However, there may be the other neural factors which are related to other object-specific attachment, as well as general object attachment. Further research on different categories of products would be helpful to achieve the generalizability of the present findings. Moreover, it is unclear, in the present study, as to whether or how the other factors or the emotions that construct “attachment” are involved in the neural activity and connectivity related to object attachment. Further research on this topic should be performed for knowing more about the meanings of the neural networks related to object attachment.

## Methods

### Participants

A total of 20 healthy right-handed women (age [mean ± standard deviation]: 33.4 ± 3.5 y) participated in this study. All recruited participants were regular users of one of the three face seram of famous luxury brands (A, B, and C), consuming them more than three times per week. These cosmetics were all within the same price range (A: \13,500/50 mL; B: \13,500/60 mL; C: \13,000/50 mL). The number of users for each serum was similar (A: n = 7; B: n = 7; C: n = 6), and more than five bottles of serum (5.2 ± 2.0 bottles) were used at the time of the fMRI experiment. Participants did not have any history of neurological or psychiatric disorders and provided written and oral informed consent to participate in the study. The Research Ethics Committee of the Shiseido Global Innovation Center approved this study, and all experiments were conducted in accordance with the relevant guidelines.

### Experimental stimuli and procedure

**Figure 7:**
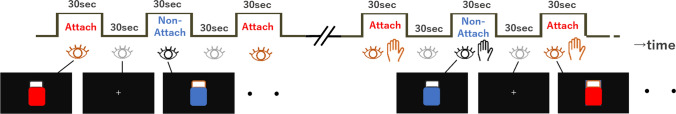
Experimental paradigm of the fMRI experiment.

We used three types of face serums (A, B, and C) that had different textures and were in bottles with different visual appearances. Each participant could clearly discriminate the serum that she regularly used from the others. The fMRI experiment consisted of two sessions (with or without tactile cues) for each participant, with two types of stimuli (attached or non-attached cosmetic) per session: 1) a visual session consisting of the serum regularly used (attached cosmetic in visual session: AV) and the control (non-attached cosmetics in visual session: nAV), repeated four times, and 2) a visual with tactile session consisting of the attached cosmetic in the visual with tactile session (AVT) and the non-attached cosmetics in the visual with tactile session (nAVT), repeated four times. Each stimulus was presented for 30 s (task block) with a 30 s interval (rest block). The stimulus presentation order was counterbalanced across participants. In the first visual session, participants viewed the photo of a face serum bottle as a stimulus in the MRI scanner using goggles that allowed the photos to be projected. In the second visual with tactile session, participants were applied with the face serum on the back of their left hands while they viewed the photo of the face serum bottle (Fig. 7). The amount of serum applied was 0.2 mL per task block. A beauty specialist applied the serum on the back of the participant’s hand using the fingers and palm of her right hand, moving her hand slowly and softly in a circle during application. The speed of movement was approximately 2.5 s per cycle. The application procedure was the same for all the task blocks. During the rest block, the other staff removed the serum from the participant’s hand using a warm wet towel. The participants were instructed to pay attention to and experience or feel the stimuli without thinking anything during the experiment.

### Subjective evaluation

After the fMRI experiment, participants evaluated all the face serums on a visual analog scale (from 0 to 100 points) of six items: “positive feeling of texture,” “expectation for skincare effect,” “want to buy,” “security,” “satisfaction,” and “attachment” after being applied with the serum in the same manner as they had in the scanner. The average scores of each subjective evaluation were compared between the attached and non-attached cosmetics using a paired *t*-test with a significance level of *p* = 0.05.

### Functional MRI data analysis

Scanning was conducted using a 3.0 T MRI system (Achieva Quasar Dual; Philips Medical Systems, Best, the Netherlands). Blood oxygenation level-dependent (BOLD) T2*-weighted magnetic resonance signals were measured using a gradient echo-planar imaging (EPI) sequence (repetition time [TR], 3,000 ms; echo time [TE], 35 ms; flip angle [FA], 90°; field of view (FOV), 230 × 230 mm^2^; scan matrix, 128 × 128; total scan time, 984 s; dynamic scans, 328 volumes; slice thickness, 5 mm; 23 slices per volume). Image processing was conducted using statistical parametric mapping software (SPM12, Wellcome Department of Imaging Neuroscience, London, United Kingdom; http://www.fil.ion.ucl.ac.uk/spm/software/spm12). T1-weighted anatomical images were acquired (TR, 23 ms; TE: 2.0 ms; FA, 30°; FOV, 240 × 240 mm^2^; scan matrix, 240 × 240; slice thickness: 1.0 mm; 150 slices). EPIs were spatially realigned, co-registered, and normalized to the Montreal Neurological Institute template. Normalized images were smoothed using an 8 mm full-width half-maximum Gaussian kernel. The data were temporally convolved with a hemodynamic response function (HRF) and high-pass filtered with a cutoff period of 128 s. The AV, nAV, AVT, and nAVT conditions were modeled using a separate regressor for the first-level analysis, and the second-level random effects analysis was performed for the contrasts: [AV vs. baseline], [nAV vs. baseline], [AVT vs. baseline], [nAVT vs. baseline], [AV vs. nAV], [AVT vs. nAVT], and [(AV + AVT) vs. (nAV + nAVT)]. For the ROI analysis, we set the brain regions that had been identified in previous fMRI studies of human love attachment in relationships^18–20,34^ (Table 1) and attachment to object (object possession)^30^ as a set of spherical ROIs (radius 5 mm). The significance of these ROIs was tested in each contrast of [AV vs. nAV] and [AVT vs. nAVT] using the SVC test (significance level: *p* = 0.05, FWE). Next, we conducted a 2 × 2 (attachment × touch) repeated measures ANOVA for the ROIs retained after the SVC test and the additional ROIs reported in previous studies^18,19,30^ at *p* < 0.05. The additional ROIs were as follows: the right accumbens/ventral striatum (0, 10, 0)^18^, the left VP (− 34, − 2, − 6) and right VP (20, − 6, − 8)^19^, and the left VP (− 9, 0, 6) and right VP (18, − 9, 3)^30^. When there was a significant interaction between attachment and touch, we compared their activities between the attached and non-attached conditions using a paired *t*-test based on a Bonferroni correction (*p* < 0.025 = 0.05/2).

Furthermore, we performed PPI analyses to identify brain regions whose activity depends on an interaction in the main effect of attachment ([AV + AVT] vs. [nAV + nAVT]). In addition, we performed PPI analysis on brain regions whose activity depends on interactions in the interactive effect of ([AVT + nAV] vs. [nAVT + AV]). For each seed ROI that showed a significant main effect of attachment or a significant interactive effect of attachment and touch in the ANOVA, the PPI procedure was performed at the single-subject level. For each participant, the seed region (5 mm radius sphere) was localized around the local maxima of the ROI. We then extracted the time course of activity in the ROI for each subject. The PPI analysis employed three regressors as follows: the deconvolved activation time course in the seed (Physiological), each of the contrasts of the main and interactive effect (Psychological), and their interaction (PPI). These regressors were entered into a first-level general linear model after deconvolution of the HRF, and contrast images of the PPI effects for each participant were entered into a random effects analysis at the second-level analysis (peak *p* = 0.005 and cluster-level pFWE < 0.05; peak *p* = 0.001 and cluster-level FWE < 0.05).

For each region that showed significant connectivity with the seed, multiple regression analyses were conducted, with individual strength of connectivity (beta value of PPI variable) as the dependent variable, and each individual subjective evaluation and individual brainstem ROI activities as the independent variables, in the main effect contrast ([AV + AVT] vs. [nAV + nAVT]. The analyses were based on a stepwise method. Furthermore, we checked the residuals for all regression analyses by performing a Kolmogorov–Smirnov test of normality and calculated the D-W statistic for the null hypothesis of no autocorrelation. The significance level was set at *p* = 0.05, for all analyses.

## Data Availability

Due to confidentiality agreements with the participants, the data in this study are available only at the Shiseido Global Innovation Center and Tokyo Metropolitan University.
